# Chemical Composition of Essential Oils of Seven *Polygonum* Species from Turkey: A Chemotaxonomic Approach

**DOI:** 10.3390/molecules27249053

**Published:** 2022-12-19

**Authors:** Azize Demirpolat

**Affiliations:** Plant and Animal Production Department, Vocational School of Food, Agriculture and Livestock, University of Bingöl, Bingöl 12000, Turkey; ademirpolat@bingol.edu.tr

**Keywords:** *Polygonum*, (E)-*β*-farnesene, dodecanal, Chemotaxonomy, GC-MS

## Abstract

Medicinal plants and herbal preparations are gaining attention in the scientific community today, as they are often used intermittently in the treatment of various diseases. The genus of *Polygonum* (Polygonaceae), known locally as “madimak”, is an aromatic plant widely used in world flavors. The chemical composition of the essential oils of dried aerial parts of seven of *Polygonum* was analyzed by GC-MS. These species are *Polygonum lapathifolium* L., *Polygonum persicaria* L., *Polygonum arenastrum* Bor., *Polygonum bellardii* All., *Polygonum arenarium* Waldst. Et Kit., *Polygonum aviculare* L., and *Polygonum cognatum* Meissn. Qualitative and quantitative differences were found in the essential oil analysis of the seven *Polygonum* species. The major compounds were determined as (*E*)-*β*-farnesene (19. 46%), dodecanal (15.92%), *β*-caryophyllene (12.95%), in *P. aviculare*; (*E)-β*-farnesene (25.00%), dodecanal (20.45%), *β*-caryophyllene (9.38%), and caryophyllene oxide (8. 26%) in *P. persicaria*; dodecanal (25.65%), caryophyllene oxide (13.35%), *β*-caryophyllene (7.95%), and *(E)-β*-farnesene (6.20%) in *P. lapathifolium*, and dodecanal (19.65%), (E)-β-farnesene (13.86%), *β*-caryophyllene (8.06%), and *α*-terpineol (7.2%) in *P. arenarium*, dodecanal (16.23%), *β*-caryophyllene (16.09%), (*E)-β*-farnesene (12.26%), caryophyllene oxide (7.94%) in *P. bellardii*, *(E)-β*-farnesene (20.75%), dodecanal (17.96%), *β*-caryophyllene (13.01%), *α*-terpineol (4.97%) in *P. arenastrum*, (*E*)-*β*-farnesene (9.49%), dodecanal (14.01%), *β*-caryophyllene (11.92%), geranyl acetate (9.49%), and undecanal (7.35%) in *P. cognatum*. This study is the most comprehensive study conducted to determine the essential oil components of *Polygonum* species. In addition, a literature review on the composition of the essential oils of these *Polygonum* taxa was performed. The essential oil components of the species in our study were revealed for the first time with this study.

## 1. Introduction

The genus *Polygonum* belonging to the Polygonaceae family includes more than 300 species spread over North and South America, Asia, North Africa, and Europe [1]. They are also widely grown on roadsides, slopes, and cultivated fields [2]. It has been reported that the leaves and young shoots are used to treat various diseases such as abdominal pain, anemia, and diabetes [3,4].

The *P. lapathifolium* L., *P. persicaria* L., *P. arenastrum* Bor., *P. bellardii* All., *P. arenarium* Waldst. Et Kit., *P. aviculare* L., and *P. cognatum* Meissn. species were the subject of this study. These species were compared morphologically, and interspecies variations emerged [5,6,7]. Therefore, this study aimed to contribute to the literature in terms of evaluating the essential oil content of the *Polygonum* species, which are widely grown in Turkey, which is rich in endemic species, to reveal alternative sources and to provide data for new biological activity research.

Edible wild plants of different species have played an important role throughout human history. In many parts of the world, *Polygonum* species are frequently consumed by local people as food, mostly fresh in foods, such as salads and meals [8]. It has been proven by several studies that the *Polygonum* species have antimicrobial, antioxidant, antifungal, diuretic, insecticide, antidiabetic, antiulcer, and anticancer activities due to the phenolic components, tannins, polyuronides, flavonoid triterpenes, glycosides, and saponins it contains [9,10,11,12,13]. According to a recent study, it was determined that the *Polygonum afghanicum* and *Polygonum aviculare* are used as animal feed [14]. It is thought that the *Polygonum* species, which is consumed as food by people, has a gastroprotective effect through its reducing power and phenolic content [9]. The clinical effects of the *Polygonum aviculare* on gingivitis were investigated, and it was concluded that the plant extracts used with the mouth rinse method could be used as a supplement in treating gingivitis [15]. 

*P. cognatum*, which is cooked and used in food, and *P. aviculare* are the preferred species among the public [16]. In addition, in meadow and pasture areas, *P. arenastrum* Bor., *P. maritimum* L., *P. pulchellum* Lois., *P. convolvulus* L., and *P. dumetorum* L. species are especially common. It was stated that the *P. lapathifolium* and *P. orientale* species of the *Polygonum* genus increase the sensitivity to light in cattle [17,18]. The antioxidant activity, total phenolic compounds, chlorophyll and carotenoids, flavonoids, and chlorophyll and carotenoids content of the leaf’s extracts of *Polygonum cognatum* Meissn. were investigated. It was further determined that water extracts had high antioxidant activity [11]. It was stated that phenolic compounds, which are also found in high amounts in malt, have allelopathic effects [19,20] and antioxidant and antimicrobial activities [11].

In recent years, people have added wild edible plant species to their food lists due to their medicinal importance and aromatic properties. The fact that the nutritional content of consumed wild vegetables is higher than that of many cultivated vegetables contributed to the increase [8,21]. Many traditional botanical medicines contain bioactive components in their essential oils. Thus, the characterization of herbal essential oils is essential [22,23]. For example, the Malaysian government has listed *P. minus* in its National Agri-Food Policy to ensure adequate supply and to strengthen the agricultural economy. *P. minus* has been recognized by the Malaysian government as an essential oil-producing crop in the Herbal Product Scheme [24]. 

Among *Polygonum* species, the roots of the *P. cuspidatum* are frequently used as a source of resveratrol. Most resveratrol capsules sold as food supplements in the United States contain *P. cuspidatum* extracts, while the rest contain red wine or red grape extracts. China is one of the leading countries that obtain trans-resveratrol from *P. cuspidatum* root extracts. Many Chinese companies obtain trans-resveratrol with varying degrees of purity [25]. In a reviewed study, 2.18% trans-resveratrol and 1.07% trans-piseit were detected in *P. cuspidatum* roots using high-speed counter-current chromatography (HSCCC) [26]. In another study, different parts of the *P. cuspidatum* plant were evaluated for resveratrol content by using high-performance liquid chromatography. It was observed that the resveratrol content of its perennial root, leaf, stem, and annual root was quite high [27].

The essential oil composition of seven species belonging to the *Polygonum* genus, which is popularly consumed among the public in many parts of the world, was determined to provide basic data for bioactivity studies. As part of the author’s ongoing research on plants and to improve knowledge on the genus *Polygonum*, this study aimed to investigate the chemical composition of seven unanalyzed taxa of *Polygonum*. The essential oils of these *Polygonum* species were analyzed for the first time in this study. This study represents the first time that such a comprehensive essential oil study has been carried out on *Polygonum* species.

## 2. Results

In the essential oil analysis of the seven *Polygonum* species, qualitative and quantitative differences were found. In the essential oils of *P. aviculare* L. and *P. cognatum* Meissn. 33 components were identified representing 88.19% and 98.87% of the oils, respectively. The aerial part of *P. aviculare* and *P. cognatum* were hydrodistilled, obtaining yields of 0.9% and 0.8 (*v*/*w*) of light yellowish oils, respectively. The aerial parts of the *P. lapathifolium* L. and *P. bellardii* All. were hydrodistilled, obtaining yields of 0.9% and 0.8% (*v*/*w*) of light yellowish oils, respectively. In the essential oils of this species, 31 and 32 components were identified representing 91.8% and 95.72% of the oils, respectively. *P. arenarium* has 34 components (0.6 *v*/*w*). In the essential oils of *P. persicaria* L. 29 components and *P. arenastrum* were identified by 31 components. Additionally, this species was representing 85.30% and 90.02% of the oils, respectively. The aerial parts of the *P. persicaria* L. obtained yields of 0.8% of yellowish oils and the aerial parts of the *P. arenastrum* obtained yields of 0.9% (*v*/*w*) of yellowish oils.

The major compounds were *(E)-β*-farnesene (19.46%), dodecanal (15.92%), and *β*-caryophyllene (12.95%), in *P. aviculare*; *(E)-β*-farnesene (25.00%), dodecanal (20.45%), *β*-caryophyllene (9.38%) and caryophyllene oxide (8.26%) in *P. persicaria*; dodecanal (25.65%), caryophyllene oxide (13.35%), *β*-caryophyllene (7.95%) and (*E*)*-β*-farnesene (6.20%) in *P. lapathifolium;* dodecanal (19.65%), (*E*)-β-farnesene (13.86%), *β*-caryophyllene (8.06%) and *α*-terpinene (7.01%) in *P. arenarium;* dodecanal (16.23%), *β*-caryophyllene (16.09%), *(E)-β*-farnesene (12.26%), caryophyllene oxide (7.94%) in *P. bellardii*; *(E)-β*-farnesene (20.75%), dodecanal (17.96%), *β*-caryophyllene (13.01%), *α*-terpineol (4.97%) in *P. arenastrum*, (*E*)*-β*-farnesene (9.49%), dodecanal (14.01%), *β*-caryophyllene (11.92%), geranyl acetate (9.49%), 2-methyl-4-pentenal (9.49%), and undecanal (7.35%) in *P. cognatum.* The compositions of seven of the *Polygonum* essential oils are listed in Table 1.

## 3. Discussion

The result of this study is important regarding the usability of dodecanal and *(E)-β*-farnesene, which are the major components of the *Polygonum* species. Ullah et al. reported that the compound dodecanal (43.29%) was found to be the major component of the *P. minus* essential oil followed by 1-decanol (15.13%), isobornyl acetate (15.13%), n-decanoic acid (5.72%) [38]. In the present study, dodecanal was found in *P. aviculare* (15.92%), *P. persicaria* (20.45%), *P. lapathifolium* (25.65%), *P. arenarium* (19.65%), *P. bellardii* (16.23%), *P. arenastrum* (17.96%), and *P. cognatum* (14.01%), respectively.

Dodecanal, which was a major component in the seven species in this study, is a tyrosinase inhibitor. Tyrosinase is the enzyme involved in the formation of melanin [39]. The increase in tyrosinase activity in cancerous cells in some cancer types has drawn attention to the importance of the use of tyrosinase in the treatment of these types of cancer. Tyrosinase is a key enzyme in the food industry in maintaining the economic value of fruits and vegetables. It is responsible for the enzymatic browning of vegetables and fruits. In this context, strong tyrosinase inhibitors are needed in agriculture and food [40,41,42]. These results present new opportunities for effective tyrosinase inhibitors. On the other hand, *(E)-β*-farnesene, which has an important place among the major components of this study, is a group of sesquiterpene isomers found in essential oils. It was reported that this compound was used by aphids to warn other individuals and to repel insects [43,44]. The determination of high levels of *(E)-β*-farnesene in the essential oil content of the studied *Polygonum* species we studied provided basic data for future studies in the field of bioactivity research.

*β*-caryophyllene is a bicyclic sesquiterpene that is abundantly detected in the essential oils of many plants such as cloves, thyme, cinnamon, rosemary, and Copaiba oil [45,46,47]. It was shown to have antioxidant, anti-inflammatory, and anti-cancer properties. A former study revealed that β-caryophyllene has remedial eventuality as a hepatoprotective effect in fibrosis [48]. In another study, *β*-caryophyllene was shown to have protective effects against liver failure in mice [49].

Moreover, *β*-caryophyllene also effectively treated cisplatin-induced nephrotoxicity and decreased the pro-inflammatory cytokine expression in rats [50]. Interestingly, the *P. minus* essential oil was set up to have a high attention of *β*-caryophyllene [51]. Previous studies have shown that essential oil has hepato-protective properties [52,53]. *P. minus* contains various medicinal values and yields high levels of essential oils, mainly composed of terpenoids, organic acids, and aliphatic aldehydes [22]. A study by Rashid et al. showed that essential oils of the *P. minus* species could protect against cisplatin-induced hepatotoxicity through the inhibition of oxidative stress, inflammation, and apoptosis. However, the *P. minus* essential oils used in higher concentration showed pro-oxidant, pro-inflammatory, and pro-apoptotic effects. Therefore, it has been recommended not to use higher doses to avoid any harmful effects [54]. *β*-caryophyllene was detected in the studied *Polygonum* species at the rates of “*P. aviculare* (12.95%), *P. persicaria* (9.38%), *P. lapathifolium* (7.95%), *P. arenarium* (8.06%), *P. bellardii* (16.09%), *P. arenastrum* (13.01%), and *P. cognatum* (11.92%)”.

The components of the essential oils from the sprouts of the *Polygonum hydropiper* were analyzed using capillary GC and GC–MS. Fifty-three components were identified, representing 91.6% of the total oils. The main constituents of the essential oils were (*E*)-*β*-farnesene (44.1%), phytol (10.8%), (*E*)-caryophyllene (9.3%), and (*E*)-nerolidol (6.9%). The essential oil from *P. hydropiper* contained a high content of sesquiterpenoids [55]. The study’s findings showed that *(E)-β*-farnesene percentages were as follows, 19.46% for *P. aviculare*, 25.00% for *P. persicaria*, 6.20% for *P. lapathifolium*, 13.86% for *P. arenarium*, 12.26% for *P. bellardii*, 20.75% for *P. arenastrum,* and 9.49% for *P. cognatum*. The fact that the main components of the *Polygonum* species collected from Turkey, which were used in this study, showed similar results to the main components of the *P. hydropiper* species collected from Japan is important for the results of the present study.

Yao et al. [56] identified that the major constituents were phellandrene (13.6%), 1-methyl-4-isopropenylbenzene (7.2%), zingiberene (4.9%), and α-thujene (4.5%) while Dung et al. [57] reported that the major components were α-humulene (7.0%, 7.1%), curcumene (2.5%, 2.5%), and α-zingiberene (2.4%, 2.2%) in the stem and leaf oils from *Polygonum hydropiper*. In this study, the major components in all *Polygonum* species were *(E)*-*β*-farnesene, dodecanal, *β*-caryophyllene, and caryophyllene oxide. 

Another study reported that the analysis of oil extracted from the leaves of the *Polygonum hydropiper* revealed the presence of the following six constituents: acetic acid (36.03%), propanoic acid, ethyl ester (18.21%), *n*-propyl acetate (20.67%), and confertifolin (22.91%) [58]. In the study of Kima et al., the main volatile components obtained from *Polygonum cuspidatum* oil as a result of GS-MS were 2-hexenal (73.36%), 3-hexen-1-ol (6.97%), n-hexanal (2.81%), and 1-penten-3-ol (2.55%) [59]. In another study, a systematic relationship was found when comparing lignans isolated from the *P. cuspidatum* and *P. aviculare* species [60]. When the results of these studies were compared with the present study, the major components were different due to the differences in the methods studied [61]. 

In a study made from the aboveground and underground parts of *Polygonum* species, it was determined that *Polygonum* extracts (*P. aviculare*, *P. cognatum*, *P. patulum*, and *P. setosum*) have an inhibitory effect against cosmetic elastase and collagenase enzymes, and that catechin and quercetin glycosides, which are found at higher rates in extracts prepared from the roots of plants, may be the compounds responsible for the effect [62]. In light of this information, within the scope of this study, it is predicted that *Polygonum* essential oils are valuable, and they can show high activity in bioactivity studies. With this preliminary study, it is seen that the *Polygonum* species are promising by increasing efforts to use them both as cosmetics and as preparations.

This study’s results indicate that the seven studied *Polygonum* species, based on two major essential oil constituents, can be classified into the following three different chemotypes in the eastern Anatolian region of Turkey: dodecanal/caryophyllene oxide chemotype (*P. lapathifolium*); dodecanal/*β*-caryophyllene chemotype (*P. bellardi and P. cognatum*) and dodecanal/(*E*)*-β*-farnesene chemotype (*P. aviculare*, *P. arenastrum*, *P. Arenarium,* and *P. persicaria*).

**Figure 1:**
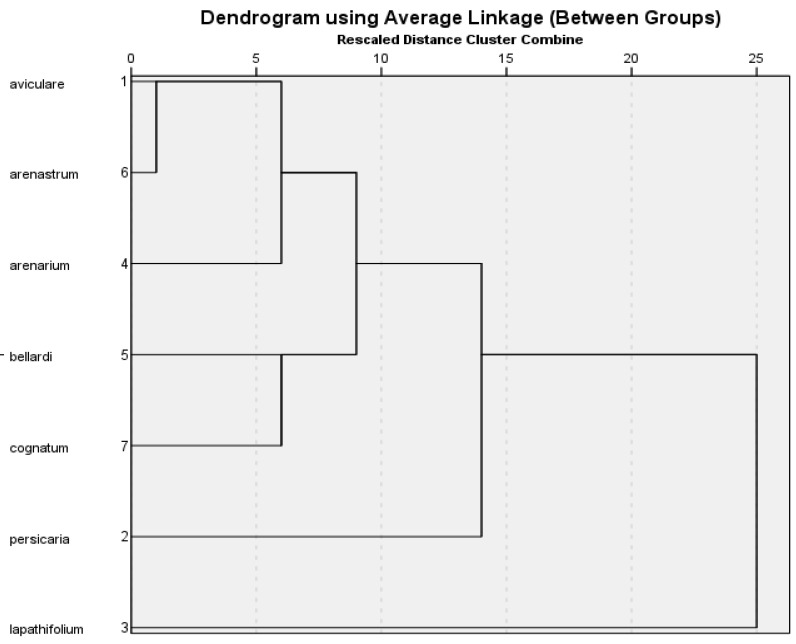
Clustering analysis of seven *Polygonum* species to essential oil components.

In the cluster analysis based on major components (≥1%), the outermost species was *P. lapathifolium* (Figure 1). Among these major components, the amounts of dodecanal and caryophyllene were the highest and the amounts of *(E)-β*-farnesene and *β*-caryophyllene were the lowest compared to the other investigated species.

*P. persicaria* was close to *P. lapathifolium* and distant to the other five species in terms of their levels of caryophyllene oxide. In addition, the *P. aviculare* and *P. arenastrum* species were the closest species in terms of chemical characteristics, they were similar, and they were included in the same clade. When the groupings were examined, it was determined that some species (*P. lapathifolium*, *P. persicaria*, *P. arenastrum*, *P. bellardii*, and *P. arenarium*) belonging to the *Polygonum* genus were compatible with the morphological description [5].

**Figure 2:**
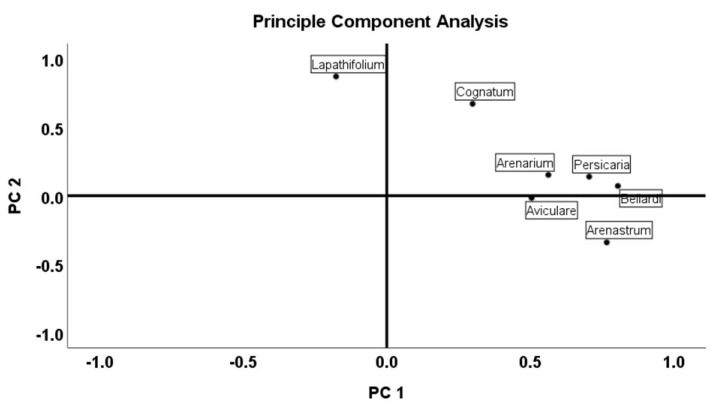
Principal component analysis (PCA) of the essential-oil composition of *Polygonum* species.

**Figure 3:**
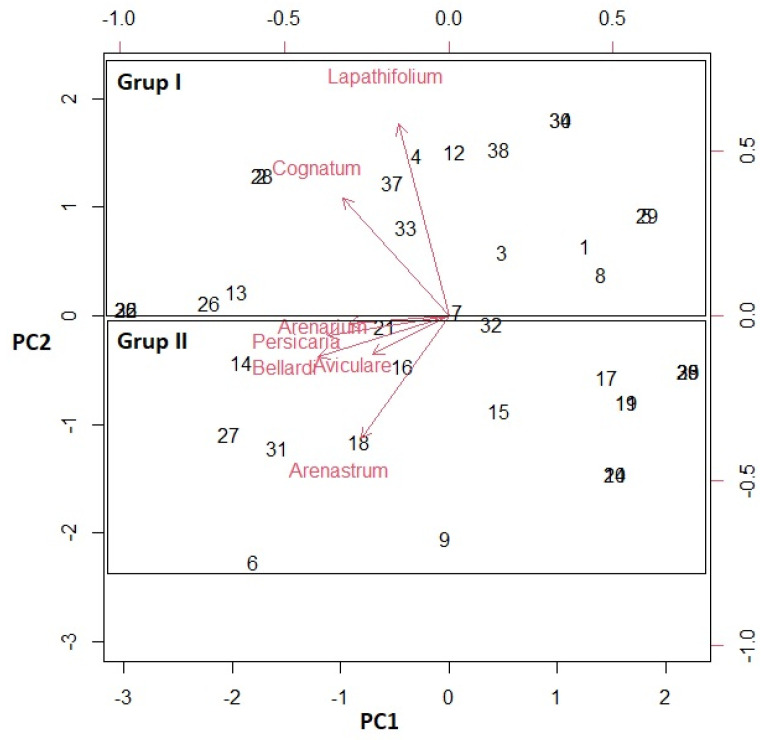
Biplot (PCA) from the analysis of the essential oil composition of the *Polygonum* species.

Multivariate analysis was used based on previously reported studies [38]. Principle component analysis (PCA) and cluster analysis (CA) were performed to identify the compounds for the different samples. The PCA was then performed with Varimax rotation using the matrix correlation configuration. The main components of the principal component analysis were PC1 with 38.19% and with PC2 16.16%, respectively. The total load of PC1 and PC2 was 54.35%. The Kaiser-Meyer-Olkin (KMO) method was conducted to examine the correlation of the variables. KMO was at 0.698, which is considered acceptable. Barlett’s test of sphericity also showed a statistical significance at alpha 0.05 for the data set. PCA analysis, which clarified the relationship between the seven *Polygonum* species and their essential oil content, was explained with two possible groups (PC1 and PC2). The results are presented in Figure 1, Figure 2 and Figure 3.

In this study, a biplot graph was constructed to determine the multivariate relationships of the compounds in the essential oils of the seven studied *Polygonum* species (Figure 3). In the biplot graph, if the angle between the vectors is less than 90°, it indicates that the content of that species is better than the average, if the angle between the vectors is greater than 90°, the content of the species is lower than the average, and if the angle is equal to 90°, it is close to the average. When Figure 3 is analyzed, it was identified which species had higher values in terms of all components and whether these features were positively or negatively related to each other. The statistical analyses supported each other.

The species included in this study belong to the same genus collected from localities close to each other. As a result, the essential oil components were positively close to each other. Concerning the chemical composition, numerous literature works have focused their investigation on the secondary metabolite constituents in both essential oils and extracts containing bioactive compounds. This study is the most comprehensive study conducted to determine the essential oil components of *Polygonum* species. The essential oil components of the species in this study were revealed for the first time in the literature.

## 4. Materials and Methods

### 4.1. Sample Collection

The *Polygonum* species were collected from natural habitats in Turkey (Table 2). Legal permission was granted from the general directorate of nature conservation and national parks to collect plants through the research permissions information system.

All the plants were collected during the flowering period and in the morning. Some of the plant material belonging to each taxon was also deposited as an herbarium sample kept at the Bingöl University Food Agriculture Livestock Vocational School. 

### 4.2. Isolation of Essential Oils and GC-MS Analysis

In this study, the plants were air-dried. The hydrodistillation method was used to obtain the oil from the plants. The air-dried aerial parts of the plant materials (200 g) were subjected to hydrodistillation for three hours using a Clevenger-type apparatus. After completion of the distillation, the organic layer in the collection vial was injected into the GC/GC-MS instrument.

The essential oil was analyzed using GC-MS. The Shimadzu GCMS-QP2020 model MS instrument were used. The ionization energy was 70 eV with a mass range of 40–330 m/z. A column RXI-5MS (30 m × 0.25 mm × 0.25 µm) column flow rate (transporter gas helium) capillary column was used. The carrier gas was helium with a 1 mL/min constant column flow rate. Column oven temperature program had the following settings: a temperature of 40 °C and a hold time two minutes at a rate of 3 °C/min. The final temperature was 240 °C. The injection volume was selected as 1 µL and the mode was selected as a split (split ratio 1:10 or 1:100). In hexane samples, a 3.5 min solvent delay was used.

The mass spectrometric parameters were as follows: full scan mode, scan speed of 20,000 amu/s, and a sampling frequency of 50 spectra. The interface and ion source temperatures were 250 °C and 200 °C, respectively.

The retention indices (RI) were calculated using alkanes as reference points. The chemical compounds of the essential oils were identified by comparing their RI to those of n-alkanes (C8–C22) as external references, their retention times (RT), and their mass spectra with that reported in MS libraries (Wiley) and in the literature (NIST 20 and Wiley Libraries) [63]. A traditional library search neglects the retention parameters, and the process only involves the comparison of spectra. A combination of storage indexes was used in this study when searching libraries, making the identification of compounds easier and more reliable. This study also used the device’s retention index spectral libraries. For better results, the same analysis method was used as the same column described in the library.

The identified constituents of the essential oils are listed in Table 1.

### 4.3. Cluster Analysis and PCA (Principal Component Analysis)

Ten major components (≥1%) were selected from the essential oil components obtained by water distillation. These components were subjected to cluster analysis from numerical taxonomic methods for the seven *Polygonum* species. For this analysis, the IBM SPSS Statistics 21.0.0 software program and the UPGMA statistical method were used. The results of these analyses were illustrated using dendrograms and evaluated in terms of numerical chemotaxonomic relationships. In the resulting cluster analysis tree, the relationships of the species are indicated (Figure 1). 

Multivariate analysis was performed to identify the structure of variability and to measure the distances between groups. These analyses were performed on complete data sets. The UPGMA (unweighted pair-group average linkage) clustering method based on Pearson distances was used to measure the similarities between each measured unit (Figure 2). The chemical components of the essential oils of the seven *Polygonum* species were used as variables. Principle component analysis (PCA) and cluster analysis (CA) were used to evaluate the compounds of different samples. The raw data were standardized to the same weight as previously reported.

The PCA was then performed with Promax rotation using the matrix correlation configuration. PCA was then performed using the matrix type correlation configuration in the SPSS software. The proximity and distance of the species from each other according to their essential oil content are discussed. Biplot graphs were made in the R software version 4.2.2 (Figure 3).

## Figures and Tables

**Table 1:** Essential oils chemical composition of seven the *Polygonum* species.

No	Component	RI	RI(lit)	RT	Identification Method	% P1	% P2	%P3	%P4	%P5	%P6	%P7
					***Monoterpenes***							
**1.**	*α*-thujene	928	935 [28]	3.481	RI, MS	1.32	0.61	1.78	0.57	-	0.84	0.39
**2.**	*α*-pinene	938	937 [29]	3.790	RI, MS	0.84	1.51	1.27	1.01	2.76	0.54	1.50
**3.**	Camphene	953	952 [30]	4.654	RI, MS	1.79	0.72	2.85	1.45	0.22	0.25	0.35
**4.**	*β*-pinene	980	982 [31]	7.905	RI, MS	3.98	0.46	2.94	2.50	0.26	0.62	2.08
**5.**	Limonene	1027	1033 [31]	8.428	RI, MS	0.71	0.56	1.15	0.84	0.47	0.58	0.06
**6.**	Sabinene	1030	1030 [22]	12.024	RI, MS	**5.48**	1.65	0.73	1.08	1.02	2.92	0.25
**7.**	*P*-cymene	1035	1028 [31]	21.879	RI, MS	1.51	-	-	1.10	-	0.55	1.18
**8.**	*β*-myrcene	1168	1065 [32]	23.290	RI, MS	0.36	0.76	0.93	0.12	0.91	0.54	2.91
**9.**	*γ*-terpinene	1117	1064 [31]	23.495	RI, MS	1.30	0.54	-	-	6.09	2.42	0.97
**10.**	Borneol	1198	1175 [32]	27.746	RI, MS	0.78	0.72	0.76	0.12	0.54	1.16	0.09
**11.**	Bornylacetate	1285	1280 [32]	28.433	RI, MS	1.09	0.67	0.78	0.12	0.34	0.92	-
**12.**	Geranyl acetate	1378	1400 [31]	29.105	RI, MS	-	-	1.50	-	**3.90**	-	**9.49**
**13.**	*α*-terpineol	1213	1210 [32]	31.879	RI, MS	1.12	0.93	2.11	1.24	2.48	4.97	1.80
**14.**	*α*-terpinene	1179	1178 [30]	33.290	RI, MS	1.82	1.71	0.44	**7.01**	1.15	0.51	1.33
**15.**	2-carene	1181	1180 [22]	30.851	RI, MS	0.37	0.54	0.49	1.25	1.28	0.86	-
**16.**	4-carene	1192	-	31.879	RI, MS	1.76	1.96	2.55	0.98	0.66	1.53	-
**17.**	Terpinolene	1198	1177 [33]	33.290	RI, MS	-	0.72	-	1.47	0.32	0.94	0.98
**18.**	*p*-cymene	1209	1226 [33]	33.495	RI, MS	2.20	-	0.94	0.28	1.29	1.44	3.54
**19.**	*o*-cymene	1229	1230 [34]	37.746	RI, MS	1.40	-	0.44	0.58	0.39	-	0.32
					***Sesquiterpene***							
**20.**	*β*-Elemene	1380	1384 [34]	38.433	RI, MS	0.20	0.78	-	0.25	0.33	0.51	0.64
**21.**	Aromadendrene	1419	1418 [34]	39.105	RI, MS	-	1.74	-	0.13	2.43	0.86	1.16
**22.**	*β*-Caryophyllene	1393	1392 [34]	39.376	RI, MS	**12.95**	**9.38**	**7.95**	**8.06**	**16.09**	**13.01**	**11.92**
**23.**	*Β*-Cubebene	1399	1325 [32]	41.716	RI, MS	0.60	-	0.04	0.02	-	-	0.06
**24.**	Linalool	1147	1103 [35]	42.224	RI, MS	0.55	0.57	-	-	-	1.09	-
**25.**	(E)-*β*-Farnesene	1454	1477 [22]	44.523	RI, MS	**19.46**	**25.00**	**6.20**	**13.86**	**12.26**	**20.75**	**9.49**
**26.**	*β*-Bisabolene	1458	1500 [22]	44.635	RI, MS	1.84	1.11	3.25	0.55	3.09	3.01	1.44
**27.**	*α*-Bisabolene	1506	1507 [32]	45.240	RI, MS	0.31	1.89	0.88	1.26	4.65	1.79	2.25
**28.**	Caryophyllene oxide	1457	1456 [32]	46.263	RI, MS	**-**	**8.26**	**13.35**	3.15	**7.94**	0.75	2.11
**29.**	*Trans-α*-bergamotol	1451	1692 [9]	48.155	RI, MS	0.74	0.34	3.40	0.86	0.50	0.52	0.82
**30.**	Humulene epoxide	1458	1415 [32]	49.972	RI, MS	0.51	0.49	3.70	0.55	0.90	-	3.35
					***Aliphatic compounds***							
**31.**	Decane, 4-methyl	1400	-		RI, MS	1.19	-	-	1.93	1.65	2.25	2.11
**32.**	Decanol	1407	1766 [36]	52.049	RI, MS	0.32	0.90	2.11	6.74	0.94	4.42	0.88
**33.**	Undecanal	1408	1617 [36]	53.242	RI, MS	0.35	-	1.88	4.50	0.50	1.23	7.35
**34.**	2-methyl-4-pentenal	1466	-	54.436	RI, MS	-	0.03	3.35	-	0.90	-	**9.49**
**35.**	2-hexen-1-ol	1470	1420 [37]	57.112	RI, MS	-	-	-	0.98	-	-	-
**36.**	Dodecanal	1477	1722 [36]	61.240	RI, MS	**15.92**	**20.45**	**25.65**	**19.65**	**16.23**	**17.96**	**14.01**
**37.**	Undecane	1598	-	61.410	RI, MS	3.15	-	1.60	0.94	3.23	0.28	1.30
**38.**	Decanal	1502	1506 [36]	62.204	RI, MS	2.18	0.30	1.58	0.50	-	-	3.25
**39.**	Pentadecane	1510	-	63.126	RI, MS	0.09	-	0.68	-	-	-	-
	**TOTAL**					**88.19**	**85.30**	**91.8**	**85.65**	**95.72**	**90.02**	**98.87**

RI: retention indices; RI(lit): retention indices literature, RI—based on retention index; MS, based on mass spectra matching; RT: eetention time. **P1: *P.* *aviculare*, P2: *P. persicaria*, P3: *P***. ***lapathifolium*, P4: *P. arenarium*, P5: *P. bellardii*, P6: *P. arenastrum*, P7: *P. cognatum.***

**Table 2:** The locality information of collected *Polygonum* species.

Taxa	Locality	Collecter
*P. aviculare*	Elazığ: Sivrice Hazar lake side, 27.08.2020, 1250 m.	A. Demirpolat 6520
*P. persicaria*	Bingöl: Entrance of Dikme village, roadside, 22.07.2020, 1600–1700 m.	A. Demirpolat 6521
*P. lapathifolium*	Bingol: South of Yelesen village, steppe, 25.07.2020, 1600–1700 m.	A. Demirpolat 6522
*P. arenarium*	Bingol: West of Dikme upland, steppe, 10.07.2020, 1600–1700 m.	A. Demirpolat 6523
*P. bellardii*	Elazığ: Vicinity of Güneytepe village, sandy and moist areas, 25.07.2020, 1250–1270 m.	A. Demirpolat 6524
*P. arenastrum*	Elazığ: Vicinity of Taşkesen village, grasslands, 05.08.2009, 980–1000 m.	A. Demirpolat 6525
*P. cognatum*	Elazığ: Gümüşkavak village grasslands, 05.08.2020, 1000–1270 m.	A. Demirpolat 6526

## Data Availability

Not applicable.

## Sample Availability

Samples of the compounds are available from the authors.
